# Response of photosynthesis and electrical reactions of wheat plants upon the action of magnetic fields in the Schumann resonance frequency band

**DOI:** 10.1080/15592324.2023.2294425

**Published:** 2023-12-26

**Authors:** Marina Grinberg, Nikolay Ilin, Yulia Nemtsova, Fedor Sarafanov, Angelina Ivanova, Alexey Dolinin, Polina Pirogova, Vladimir Vodeneev, Evgeny Mareev

**Affiliations:** a Department of Biophysics, Lobachevsky State University of Nizhny Novgorod, Nizhny Novgorod, Russia; b Department of Geophysical Research, Gaponov-Grekhov Institute of Applied Physics of the Russian Academy of Sciences, Nizhny Novgorod, Russia

**Keywords:** Extremely low frequency magnetic field, Schumann resonance, light-induced electric reactions, photosynthesis, wheat

## Abstract

Alternating magnetic fields (MF) with Schumann resonance frequencies accompanied the development of living organisms throughout evolution, but today it remains unclear whether they can have a special biological effect in comparison with surrounding non-resonant frequencies. This work shows some stimulating effect of extremely low-frequency MFs on morphometric parameters and the activity of physiological processes in wheat (*Triticum aestivum* L.). It is shown that the MF effect is more pronounced for transient processes – photosynthesis reactions and changes in electrical potential caused by turning on light. For light-induced electrical reactions, the dependence of the severity of the effect on the frequency of the applied MF was demonstrated. It is shown that the most pronounced effect occurs in the 14.3 Hz field, which corresponds to the second harmonic of the Schumann resonance. The predominant sensitivity of signal-regulatory systems gives reason to assume the influence of MFs with Schumann resonance frequencies on the interaction of plants with environmental factors under conditions of a changed electromagnetic environment. Such conditions can occur, for example, with an increase in lightning activity caused by climate change, which serves as the basis for the generation of Schumann resonances, and with the development of artificial ecosystems outside the Earth’s atmosphere.

## Introduction

In the electromagnetic field frequency spectrum, there are special bands of interest, which, on the one hand, due to various reasons, can change in natural conditions, on the other hand, have a pronounced impact on the state of living organisms.^1–5^ One such band is the extremely low-frequency (ELF) Schumann resonance fields. Schumann resonances are electromagnetic oscillations of the Earth-ionosphere resonator, excited by lightning discharges. These oscillations are permanently present in the atmosphere, as they are supported by global lightning activity. The characteristic frequencies of the first, second and third Schumann harmonics are 7.8, 14.3, 20.8 Hz, respectively.^6–8^ The search for effects associated with natural resonances of magnetic fields (MFs) in living organisms is interesting in evolutionary and ecological aspects, since the severity of the spectral band associated with Schumann resonances can be modified due to its dependence on global lightning activity under climate change conditions.^9–11^ In addition, a radical change in the usual electromagnetic environment, including the disappearance or change of Schumann resonances, will take place for artificial ecosystems planned to be created at space stations and bases on other planets.^12^

Taking into account the fact that living organisms have been accompanied with the Schumann resonance frequency field throughout the entire period of evolution, it has been suggested that they developed a special sensitivity to such frequencies.^3^ To date, individual evidence has been obtained that MFs with frequencies close to the first and second harmonics of the Schumann resonance can have a more pronounced effect on living organisms compared to the neighboring band of the electromagnetic spectrum.^3,13–15^ The frequency dependence in the Schumann resonance band was studied in most detail on rat cardiomyocytes.^14^ The results indicate that the 7.8 Hz field (the first harmonic of the Schumann resonance) causes the occurrence of intracellular calcium waves and cell contraction, which does not occur when exposed to the field with other frequencies.

We have previously shown that an increased level of MF with the frequency of 14.3 Hz (the second harmonic of the Schumann resonance) can affect photosynthesis, electrical reactions, as well as drought resistance in wheat plants.^16–19^ Previous studies demonstrate that the effect of ELF MF is most pronounced during biological transient processes (e.g., the transition from a dark-adapted to a light-adapted state),^16^ including the development of stress responses (drought-induced responses).^17^ In this paper, we checked whether these effects are associated with the resonant frequency or may be explained by an increased level of ELF MF in general.

## Objects and methods

### Growth and exposure of plants in the magnetic field

The experiments were carried out on wheat plants (*Triticum aestivum* L.) variety “Daria”. Plants were grown in containers with universal soil at a temperature of 24°C and a 16-hour light/8-hour dark cycle. Lighting was provided by white and fluora fluorescent lamps.

**Figure 1.**
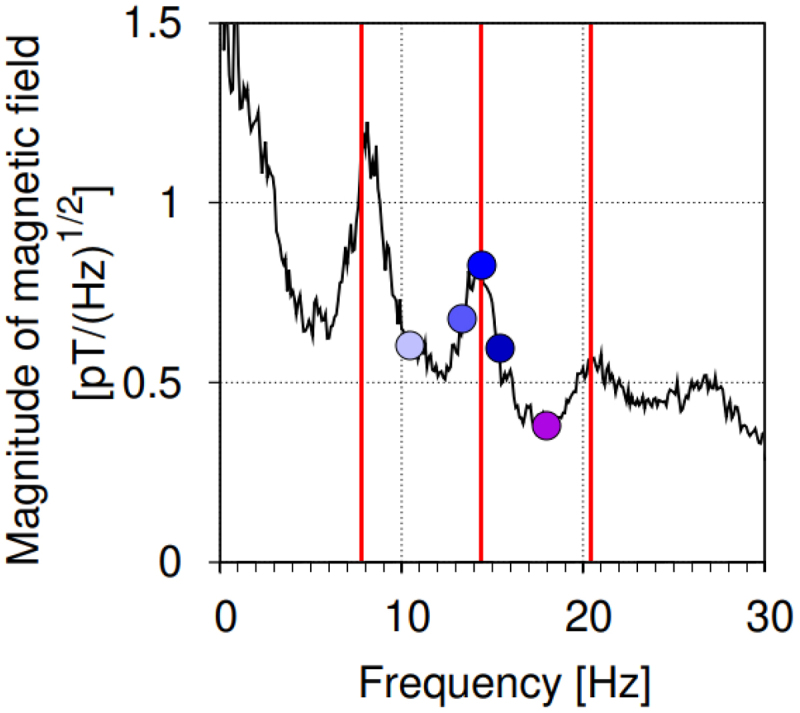
Frequency spectrum of ELF MF based on.^6^ The red lines indicate the frequencies of the first, second and third harmonics of the Schumann resonance − 7.8, 14.3 and 20.3 Hz. The blue dots indicate the frequencies at which the experiment was performed: the second harmonic of the Schumann resonance (14.3 Hz), its neighborhood (13.3 and 15.3 Hz) and the regions between resonances (10.5 and 18 Hz). The color coding of the points corresponds to that in the following figures.

Alternating magnetic fields were set by Helmholtz coils in accordance with previous works.^16–19^ The component of the induction of the Earth’s magnetic field located parallel to the alternating field in the coils was 19 μT, the component perpendicular to the alternating field was 9 μT, and the vertical component was 33 μT. The experiment was carried out in parallel at several field frequencies corresponding to the second Schumann resonance and its neighborhood: 10.5, 13.3, 14.3, 15.3 Hz (Figure 1), as well as in the absence of an additional field (Control). For experiments with light-induced electrical responses, a frequency of 18 Hz was also used. The choice of the second harmonic neighborhood is due to the large biological effect obtained in previous works.^18^ The field magnitude at all frequencies was 18 μT, which corresponded to the magnitudes used in our experiments previously.^17,18^ The plants were exposed to the field throughout the entire growing period.

## Methods

### Registration of morphometric parameters

The length, fresh and dry weight of shoots and roots were measured on the 24th day of plant growth. To measure dry weight, plants were dried in two heating cycles lasting 3 hours at 100°C.

### Registration of chlorophyll fluorescence parameters

To record the dynamics of chlorophyll fluorescence parameters, reflecting the activity of photosynthesis, an Imaging-PAM MINI PAM fluorimeter (Heinz Walz GmbH, Germany) was used. Photosynthetic parameters were calculated using the built-in software of the device.^20^ Saturation flashes were delivered at a frequency of once every 30 s. The photon flux density of actinic light (480 nm) was 223 μmol m^−2^s^−1^.

Registration of photosynthesis activity was carried out on 14–15-day-old plants. The measurements were preceded by dark adaptation lasting 20 min. The illumination period lasting 15 min. The range of 5.5–6 min after light on was used to calculate photosynthetic rates during transient processes. The measurements were made under the same magnetic field conditions under which the plants were grown.

### Registration of light-induced electric reactions

Surface potentials were measured using Ag^+^/AgCl macroelectrodes located on the second leaves of plants at a distance of 7–10 cm from the tip of the leaf. The measuring electrodes were in contact with the leaves through threads wetted with a standard solution (0.5 mM NaCl, 1 mM KCl, 0.5 mM CaCl_2_). The reference electrode was in contact with the roots through wet soil. Data were recorded using a high-impedance amplifiers IPL-113 (Semico, Russia) and processed on a PC in the param2 program.

Registration of light-induced electrical reactions was carried out on 14–15-day-old plants. To record light-induced reactions, plant leaves were illuminated using a LED matrix (455 nm) with an intensity of 625 µmol m^−2^s^−1^. A part of leaf with measuring electrode was illuminated. The measurements were preceded by dark adaptation lasting 40 min. The illumination period lasted 40 min. The amplitude of the light-induced reaction was recorded at 40 minutes after the light was turned on. The measurements were made under the same magnetic field conditions under which the plants were grown.

### Statistics

Each series of experiments consisted of 30–60 repetitions; every replicate was performed on a separate plant. Statistical analysis was performed using GraphPad Prism 6 software. The mean and standard error of mean (SE) were calculated, and the normal data distribution was confirmed for all the experiments; the significance of differences was evaluated by Student t-test. Typical and averaged records are also presented in the results.

## Results

### Effect of magnetic field on morphometric parameters

**Figure 2.**
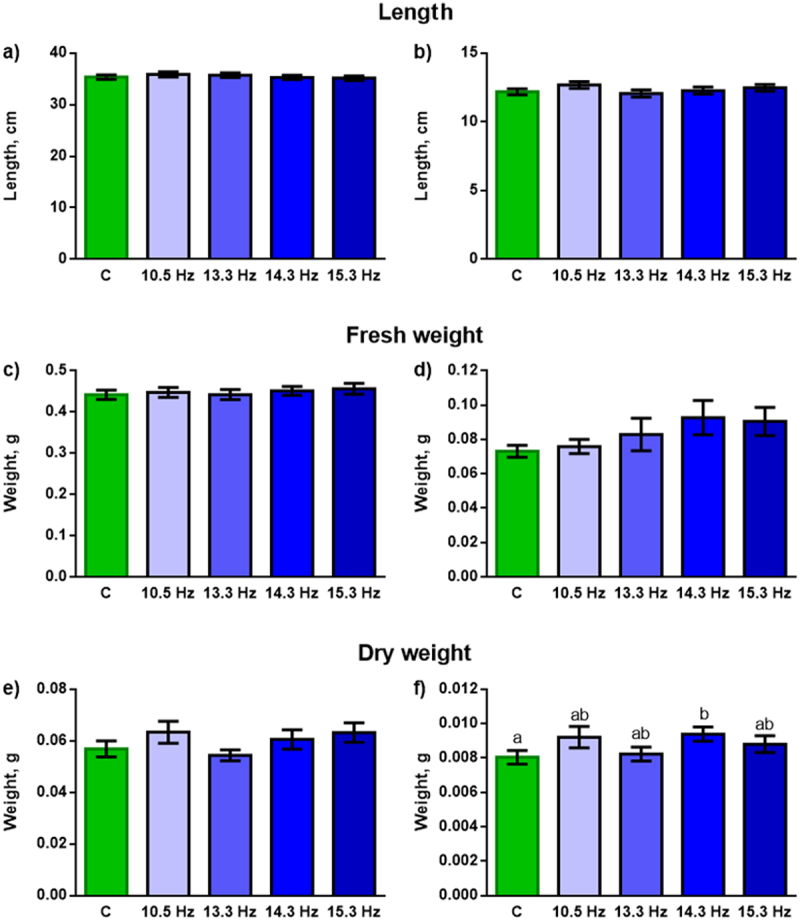
Effect of magnetic fields with frequencies of 10.5, 13.3, 14.3 and 15.3 Hz on the morphometric parameters of wheat plants. (a) and (b) – length of leaves and roots, respectively, (c) and (d) – fresh weight of leaves and roots, respectively, (e) and (f) – dry weight of leaves and roots, respectively. C – plants grown under control conditions without an additional ELF MF. *n* = 60.

To assess the integral influence of magnetic fields with different frequencies on the state of plants, their morphometric parameters, such as length, fresh and dry weight of leaves and roots, were recorded. There was practically no statistically significant effect of ELF MF on morphometric parameters. Only a slight increase in root dry weight was shown in plants grown under a field with a frequency of 14.3 Hz (second harmonic of the Schumann resonance) (Figure 2). Statistically significant differences were not revealed when comparing the parameters of control plants with those of plants exposed to other (non-resonant) frequencies. There were also no differences among all field treatments (Figure 2).

### Effect of magnetic field on chlorophyll fluorescence parameters

**Figure 3.**
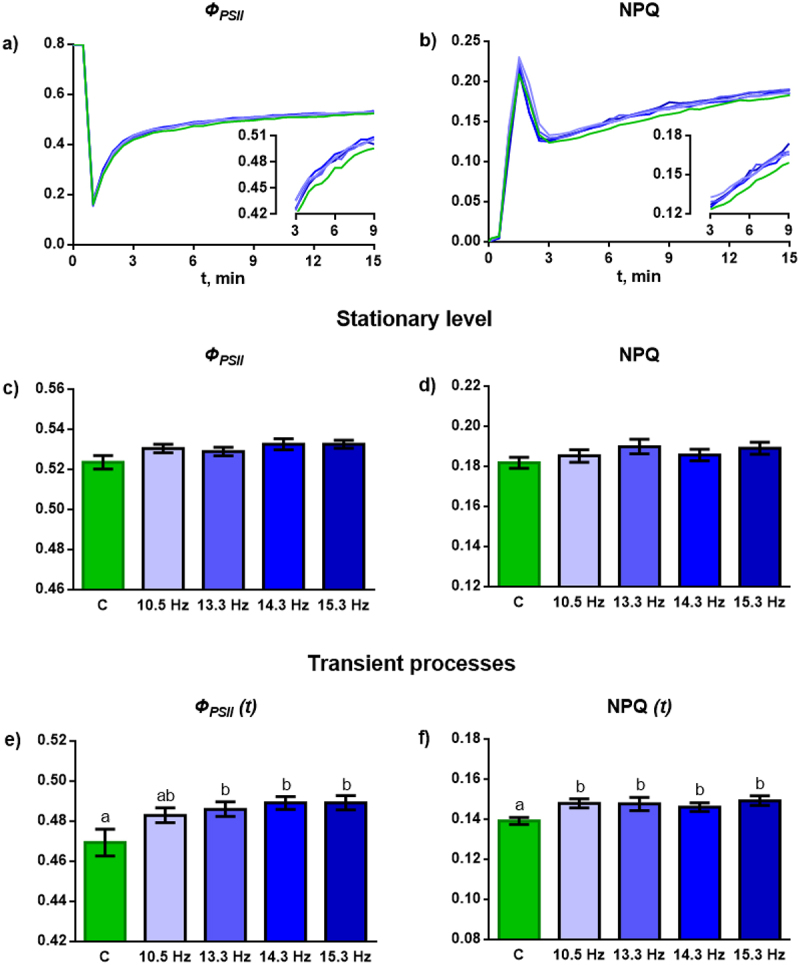
Effect of magnetic fields with frequencies of 10.5, 13.3, 14.3 and 15.3 Hz on the chlorophyll fluorescence parameters in wheat plants. (a) and (b) – averaged records of the dynamics of Ф_PSII_ and NPQ during the transition from a dark-adapted state to a light-adapted state. The inserts show fragments of the recording with transient processes in the range of 3–9 minutes. (c) and (d) – level of Ф_PSII_ and NPQ in a light-adapted (stationary) state. (e) and (f) – level of Ф_PSII_ and NPQ during the transition from a dark-adapted state to a light-adapted state. Ф_PSII_ – quantum yield of photosystem II. NPQ – non-photochemical quenching of chlorophyll fluorescence. C – plants grown under control conditions without an additional ELF MF. The color coding for graphs (a) and (b) corresponds to that for graphs (c) - f). *n* = 36.

To assess the effect of MFs with different frequencies on the intensity of physiological processes, chlorophyll fluorescence parameters, which reflect the activity of photosynthesis, were recorded. The maximum (F_v_/F_m_) and effective (Ф_PSII_) quantum yield of photosystem II and non-photochemical quenching of chlorophyll fluorescence (NPQ) were considered as the main parameters. In response to turning on the light, wheat plants develop a light-induced reaction, during which Ф_PSII_ and NPQ increase non-monotonically and reach a light-adapted level (Figure 3). ELF MF did not have a statistically significant effect on the F_v_/F_m_ value. In the light-adapted state, Ф_PSII_ and NPQ showed a weak, statistically insignificant tendency to increase under the influence of the field at all frequencies (Figure 3). No differences were found among field treatments with different frequencies.

The influence of ELF MF was revealed for the transition process – the intermediate stage of development of the light-induced photosynthetic reaction before the parameters reach a stationary level. ELF MF promotes an increase in the levels of Ф_PSII_ and NPQ compared to the control in the first minutes after turning on the actinic light. MF with frequencies of 13.3, 14.3 and 15.3 Hz significantly increases the level of Ф_PSII_, MF with a frequency of 10.5 Hz causes its increase as a trend. The NPQ level was significantly increased for all treatments. No frequency dependence was found for Ф_PSII_ and NPQ during transient processes.

### Effect of magnetic field on light-induced electric reaction

**Figure 4.**
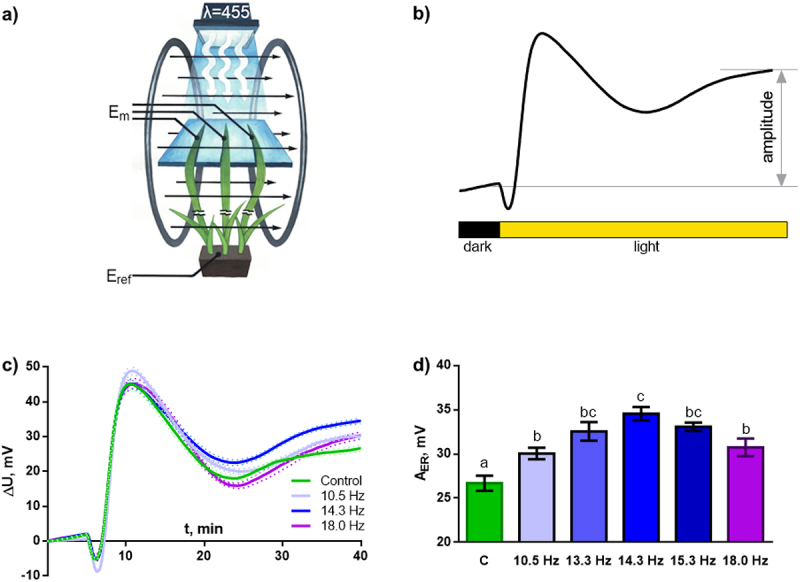
Effect of magnetic fields with frequencies of 10.5, 13.3, 14.3, 15.3 and 18 Hz on the parameters of light-induced electrical reactions of wheat plants. (a) – scheme for recording light-induced electrical reactions. Illumination was produced with blue light (455 nm) using an LED matrix. Measuring electrodes (E_m_) were located on the illuminated areas of the plant leaf. The reference electrode was located in the root area without light. (b) – scheme for calculating the amplitude of light-induced electrical reactions of wheat plants. (c) – averaged recordings of light-induced electrical reactions under MF with frequencies of 10.5, 14.3 and 18 Hz. The dotted line indicates standard error of mean (SE). (d) – amplitudes of light-induced electrical reactions (A_ER_) under MF with frequencies of 10.5, 13.3, 14.3, 15.3 and 18 Hz. C – plants grown under control conditions without an additional ELF MF. *n* = 30.

To assess the influence of MFs with different frequencies on the parameters of electrical reactions, their amplitude was recorded in response to turning on the light. The experiment showed that all treatments significantly increase the amplitude of the second, long-lasting wave of hyperpolarization caused by light (Figure 4) and do not affect the remaining stages of the development of the light-induced reaction. A dependence of the severity of the MF effect on frequency was discovered. It is shown that the amplitude at the resonant frequency of 14.3 Hz significantly exceeds the amplitudes at frequencies of 10.5 and 18 Hz, located in the spectrum minima between the Schumann resonances (Figure 1).

## Discussion

Chronic environmental factors, which in particular include ELF MF, cause long-term changes in plant development,^1,4,5,18,21–23^ The presence of such changes can be registered by integral growth parameters and the activity of basic physiological processes. In our experiments, we saw virtually no effect of ELF MF on the morphometric parameters of wheat plants. A weak stimulating effect was observed only for root weight (Figure 2). According to the literature, growth stimulation is a characteristic response of plants to the action of low-intensity MFs of various frequencies, however, a pronounced effect, as a rule, occurs for fields of higher intensity.^1,4,5,22,23^

The effect on morphometric parameters is based on changes in the activity of physiological processes, primarily photosynthesis. Our work did not reveal a pronounced effect of ELF MF on the F_v_/F_m_ value, as well as on the Ф_PSII_ and NPQ values in a stationary (light-adapted) state (Figure 3). Apparently, the effect on chlorophyll fluorescence parameters, as well as on morphometric parameters, is exerted by fields of higher intensity.^4,21,23^ Transient processes of photosynthesis, like other transient processes in the plant organism, demonstrate greater sensitivity to ELF MF.^16–18^ However, the magnitude of the identified differences in the level of Ф_PSII_ and NPQ is so small that it does not allow us to study the frequency dependence (Figure 3).

The light-induced electrical reaction is the only process studied in the experiment whose parameters change significantly under the ELF MF. Stimulation, like our previous work,^16^ was demonstrated for the second wave of hyperpolarization, the longest (main) stage of the light-induced electrical reaction (Figure 4). A significant increase in electrical reactions is shown at all frequencies used in the experiment. It was found that the severity of the influence of the field on the parameters of electrical reactions depends on the applied frequency. The greatest increase in the amplitude of reactions was recorded for the Schumann resonance frequency of 14.3 Hz, the smallest for frequencies of 10.5 and 18 Hz, which are at the minimum of the spectrum (Figure 1). Thus, the experiment may indicate the presence of resonance for the electrical reactions of plants.

The nature of the dependence of the effect on the ELF MF frequency, which was observed in our experiments, is a controversial issue. Moreover, there is not enough data in the literature to unambiguously build a consistent scheme of the influence of any MF on physiological processes. Ca^2+^, ROS and blue light receptors cryptochromes (or other flavoprotein phosphoreceptors) are the most likely participants in plant signaling cascades simultaneously involved in the formation of a light-induced electrical reaction^24–27^ and in the perception of MF^1,28–31, 32^ One of the likely participants in the formation of frequency dependence seems to be Ca^2+^ ions. Our previous work directly demonstrated that the amplitude of light-induced electrical reactions depends on the Ca^2+^ concentration under MF with a frequency of 14.3 Hz.^16^ The significant role of Ca^2+^ ions in responses to MFs with Schumann resonance and its neighborhood frequencies was also shown in a number of studies performed on animal cell cultures.^3,13–15,33^ These works show that the ELF MF-induced effect (including calcium response) may be absent at frequencies located far from resonance. For a possible explanation of this phenomenon, a mechanism is sometimes invoked that is associated with a change in the activity of Ca^2+^-binding proteins as the frequency of the alternating field approaches the frequency of the calcium cyclotron resonance at a given constant field value.^33–36^ The cyclotron resonance frequency of calcium and its subharmonics can be calculated using the formula $f=\frac{1}{n}\frac{q}{2\pi \;m}B_{DC}$, where n – is the subharmonic number, q – is the ion charge, m – is the ion mass, B_DC_ – is the induction of a constant geomagnetic field. Estimates show that at mid-latitudes of the Earth, the horizontal field component, which is approximately 20 μT, can provide a subharmonic frequency of the calcium cyclotron resonance, close to the frequencies of the Schumann resonances.

To explain the resonant effects under ELF MF, alternative hypotheses are also proposed. In particular, it is proposed to consider the mechanism of radical pairs in relation to the cell’s own periodic processes, such as the transport of electrons along the electron transport chains of mitochondria, the frequency of which can coincide with the frequency of the applied alternating field.^37^ It can also be assumed that the very structure of ion channels or components of signaling systems that can influence their permeability, due to currently unknown mechanisms, has selective sensitivity to individual MF frequencies. This mechanism may be indirectly indicated by the change in the conductivity of voltage-dependent sodium and potassium channels discovered in experiments on mouse neurons under the influence of a field with a frequency close to the second harmonic of the Schumann resonance.^38^ Additional research should clarify the correctness of these assumptions.

## Conclusion

Thus, our experiments show some stimulating effect of MFs with Schumann resonance frequencies on morphometric parameters, photosynthetic activity and the amplitude of light-induced electrical reactions in wheat plants. However, the ELF MF effect on morphometric parameters and chlorophyll fluorescence parameters was very weak, which did not allow us to study their frequency dependence. For electrical reactions, a dependence of the severity of the effect on the frequency of the applied MF was discovered. It is shown that the most pronounced effect occurs at a frequency of 14.3 Hz, which corresponds to the second harmonic of the Schumann resonance. Taken together with the data presented in the literature with similar effects for cardiomyocytes and neurons in animals, the obtained result may indicate the universality of the effects of MFs with Schumann resonance frequencies for various biological taxa. The predominant sensitivity of signal-regulatory systems gives reason to assume the influence of MFs with Schumann resonance frequencies on the interaction of living organisms with environmental factors under conditions of a changed electromagnetic environment. Such conditions can occur, for example, with an increase in thunderstorm activity caused by climate change, which serves as the basis for the generation of Schumann resonances, and with the development of artificial ecosystems outside the Earth’s atmosphere.

## References

[1] Maffei ME. Magnetic field effects on plant growth, development, and evolution. Front Plant Sci. 2014 Sep 4;5:445. doi:10.3389/fpls.2014.00445. 25237317 PMC4154392

[2] Lai H. Exposure to Static and Extremely-Low Frequency Electromagnetic Fields and Cellular Free Radicals. Electromagn Biol Med. 2019;38(4):231–7. doi:10.1080/15368378.2019.1656645. 31450976

[3] Price C, Williams E, Elhalel G, Sentman D. Natural ELF fields in the atmosphere and in living organisms. Int J Biometeorol. 2021 Jan;65(1):85–92. doi:10.1007/s00484-020-01864-6. 32034466

[4] Radhakrishnan R. Magnetic field regulates plant functions, growth and enhances tolerance against environmental stresses. Physiol Mol Biol Plants. 2019;25(5):1107–1119. doi:10.1007/s12298-019-00699-9. 31564775 PMC6745571

[5] Sarraf M, Kataria S, Taimourya H, Santos LO, Menegatti RD, Jain M, Ihtisham M, Liu S. Magnetic Field (MF) applications in plants: an overview. Plants. 2020;9(9):1139. doi:10.3390/plants9091139. 32899332 PMC7570196

[6] Ando Y, Hayakawa M. Recent studies on Schumann resonance. IEEJ Trans FM. 2006;126(1):28–30. doi:10.1541/ieejfms.126.28.

[7] Heckman SJ, Williams E, Boldi B. Total global lightning inferred from Schumann resonance measurements. J Geophys Res: Atmos. 1998;103(D24):31775–31779. doi:10.1029/98JD02648.

[8] Nickolaenko A, Hayakawa M. Schumann resonance for tyros. Springer geophysics. Tokyo: Springer; 2014. doi:10.1007/978-4-431-54358-9.

[9] Bekenshtein R, Price C, Mareev E. Is Amazon deforestation decreasing the number of thunderstorms over South America? Q J Roy Meteor Soc. 2023;149(755):2514–2526. doi:10.1002/qj.4518.

[10] Sekiguchi M, Hayakawa M, Nickolaenko AP, Hobara Y. Evidence on a link between the intensity of Schumann resonance and global surface temperature. Ann Geophys. 2006;24(7):1809–1817. doi:10.5194/angeo-24-1809-2006.

[11] Williams E, Mareev E. Recent progress on the global electrical circuit. Atmos Res. 2014;135–136:208–227. doi:10.1016/j.atmosres.2013.05.015.

[12] Pechony O, Price C. Schumann resonance parameters calculated with a partially uniform knee model on Earth, Venus, Mars, and titan. Radio Sci. 2004 Oct;39(5):1–10. doi:10.1029/2004RS003056.

[13] Blackman CF, Benane SG, Rabinowitz JR, House DE, Joines WT. A role for the magnetic field in the radiation-induced efflux of calcium ions from brain tissue in vitro. Bioelectromagnetics. 1985;6(4):327–337. doi:10.1002/bem.2250060402. 3836676

[14] Elhalel G, Price C, Fixler D, Shainberg A. Cardioprotection from stress conditions by weak magnetic fields in the Schumann resonance band. Sci Rep. 2019 Feb 7;9(1):1645. doi:10.1038/s41598-018-36341-z. 30733450 PMC6367437

[15] Yuan Y, Wei L, Li F, Guo W, Li W, Luan R, Lv A, Wang H. Pulsed magnetic field induces angiogenesis and improves cardiac function of surgically induced infarcted myocardium in sprague-dawley rats. Cardiology. 2010;117(1):57–63. doi:10.1159/000321459. 20924179

[16] Grinberg M, Mudrilov M, Kozlova E, Sukhov V, Sarafanov F, Evtushenko A, Ilin N, Vodeneev V, Price C, Mareev E. Effect of extremely low-frequency magnetic fields on light-induced electric reactions in wheat. Plant Signal Behav. 2022 Dec 31;17(1):2021664. doi: 10.1080/15592324.2021.2021664. 34994282 PMC9176247

[17] Mshenskaya NS, Grinberg MA, Kalyasova EA, Vodeneev VA, Ilin NV, Slyunyaev NN, Mareev EA, Sinitsyna YV. The effect of an extremely low-frequency electromagnetic field on the drought sensitivity of wheat plants. Plants (Basel). 2023 Feb 13;12(4):826. doi:10.3390/plants12040826. 36840174 PMC9963552

[18] Sukhov V, Sukhova E, Sinitsyna Y, Gromova E, Mshenskaya N, Ryabkova A, Lin N, Vodeneev V, Маreev E, Price C. Influence of magnetic field with Schumann resonance frequencies on photosynthetic light reactions in wheat and pea. Cells. 2021 Jan 13;10(1):149. doi:10.3390/cells10010149. 33451018 PMC7828558

[19] Sukhova E, Gromova E, Yudina L, Kior A, Vetrova Y, Ilin N, Mareev E, Vodeneev V, Sukhov V. Change in H^+^ transport across thylakoid membrane as potential mechanism of 14.3 Hz magnetic field impact on photosynthetic light reactions in seedlings of wheat (*Triticum aestivum* L.). Plants (Basel). 2021 Oct 18;10(10):2207. doi:10.3390/plants10102207. 34686016 PMC8537839

[20] Maxwell K, Johnson GN. Chlorophyll fluorescence — a practical guide. J Exp Bot. 2000;51(345):659–668. doi:10.1093/jexbot/51.345.659. 10938857

[21] De Souza-Torres A, Sueiro-Pelegrín L, Zambrano-Reyes M, Macías-Socarras I, González-Posada M, García-Fernández D. Extremely low frequency non-uniform magnetic fields induce changes in water relations, photosynthesis and tomato plant growth. Int J Radiat Biol. 2020;96(7):951–957. doi:10.1080/09553002.2020.1748912. 32369405

[22] Jedlička J, Paulen O, Ailer Š. Research of effect of low frequency magnetic field on germination, growth and fruiting of field tomatoes//Acta horticulturae et regiotecturae. Acta Hortic et Regiotectuare. 2015;18(1):1–4. doi:10.1515/ahr-2015-0001.

[23] Kornarzyński K, Dziwulska-Hunek A, Kornarzyńska-Gregorowicz A, Sujak A. Effect of electromagnetic stimulation of amaranth seeds of different initial moisture on the germination parameters and photosynthetic pigments content. Sci Rep. 2018;8(1):14023. doi:10.1038/s41598-018-32305-5. 30232352 PMC6145884

[24] Kim HY, Coté GG, Crain RC. Effects of light on the membrane potential of protoplasts from samanea saman pulvini: involvement of K channels and the H -ATPase. Plant Physiol. 1992 Aug;99(4):1532–1539. doi:10.1104/pp.99.4.1532. 16669070 PMC1080659

[25] Marten I, Deeken R, Hedrich R, Roelfsema MR. Light-induced modification of plant plasma membrane ion transport. Plant Biol (Stuttg). 2010 Sep;12(Suppl 1):64–79. doi:10.1111/j.1438-8677.2010.00384.x. 20712622

[26] Shabala S, Newman II. Light-induced changes in hydrogen, calcium, potassium, and chloride ion fluxes and concentrations from the mesophyll and epidermal tissues of bean leaves. Understanding the ionic basis of light-induced bioelectrogenesis. Plant Physiol. 1999 Mar;119(3):1115–1124. doi:10.1104/pp.119.3.1115. 10069851 PMC32094

[27] Zivanović BD, Pang J, Shabala S. Light-induced transient ion flux responses from maize leaves and their association with leaf growth and photosynthesis. Plant, Cell & Environ. 2005 Mar;28(3):340–352. doi:10.1111/j.1365-3040.2005.01270.x. 16021786

[28] Evans EW, Dodson CA, Maeda K, Biskup T, Wedge CJ, Timmel CR. Magnetic field effects in flavoproteins and related systems. Interface Focus. 2013;3(5):20130037. doi:10.1098/rsfs.2013.0037. 24511388 PMC3915827

[29] Hore PJ, Mouritsen H. The radical-pair mechanism of magnetoreception. Annu Rev Biophys. 2016 Jul 5;45(1):299–344. doi: 10.1146/annurev-biophys-032116-094545. 27216936

[30] Albaqami M, Hammad M, Pooam M, Procopio M, Sameti M, Ritz T, Ahmad M, Martino CF. Arabidopsis cryptochrome is responsive to radiofrequency (RF) electromagnetic fields. Sci Rep. 2020 Jul 9;10(1):11260. doi:10.1038/s41598-020-67165-5. 32647192 PMC7347919

[31] Fiorillo A, Parmagnani AS, Visconti S, Mannino G, Camoni L, Maffei ME. 14-3-3 proteins and the plasma membrane H^+^-ATPase are involved in maize (*Zea mays*) magnetic induction. Plants (Basel). 2023 Aug 7;12(15):2887. doi:10.3390/plants12152887. 37571041 PMC10421175

[32] Maeda K, Robinson AJ, Henbest KB, Hogben HJ, Biskup T, Ahmad M, Schleicher E, Weber S, Timmel CR, Hore PJ Magnetically sensitive light-induced reactions in cryptochrome are consistent with its proposed role as a magnetoreceptor. Proc Natl Acad Sci U S A. 2012 Mar 27;109(13):4774–4779. doi: 10.1073/pnas.1118959109. 22421133 PMC3323948

[33] Foletti A, Ledda M, De Carlo F, Grimaldi S, Lisi A. Calcium ion cyclotron resonance (ICR), 7.0 Hz, 9.2 microT magnetic field exposure initiates differentiation of pituitary corticotrope-derived AtT20 D16V cells. Electromagn Biol Med. 2010 Aug;29(3):63–71. doi:10.3109/15368378.2010.482480. 20707641

[34] Fixler D, Yitzhaki S, Axelrod A, Zinman T, Shainberg A. Correlation of magnetic AC field on cardiac myocyte Ca(2+) transients at different magnetic DC levels. Bioelectromagnetics. 2012 Dec;33(8):634–40. doi:10.1002/bem.21729. 22532275

[35] Lednev VV. Possible mechanism for the influence of weak magnetic fields on biological systems. Bioelectromagnetics. 1991;12(2):71–5. doi:10.1002/bem.2250120202. 2039557

[36] Pazur A, Rassadina V. Transient effect of weak electromagnetic fields on calcium ion concentration in arabidopsis thaliana. BMC Plant Biol. 2009 Apr 30;9(1):47. doi: 10.1186/1471-2229-9-47. 19405943 PMC2681476

[37] Krylov VV, Osipova EA. Molecular biological effects of weak low-frequency magnetic fields: frequency-amplitude efficiency windows and possible mechanisms. Int J Mol Sci. 2023 Jul 1;24(13):10989. doi:10.3390/ijms241310989. 37446167 PMC10342092

[38] Zheng Y, Dou JR, Gao Y, Dong L, Li G. Effects of 15 Hz square wave magnetic fields on the voltage-gated sodium and potassium channels in prefrontal cortex pyramidal neurons. Int J Radiat Biol. 2017 Apr;93(4):449–455. doi:10.1080/09553002.2016.1259671. 27924669

